# Proteomic profiling of regenerated urinary bladder tissue in a non-human primate augmentation model

**DOI:** 10.1038/s41598-024-66088-9

**Published:** 2024-07-09

**Authors:** Tiffany T. Sharma, Seby L. Edassery, Nachiket Rajinikanth, Vikram Karra, Matthew I. Bury, Arun K. Sharma

**Affiliations:** 1 Division of Pediatric Urology, Ann and Robert H. Lurie Children’s Hospital, Chicago, IL 60611 USA; 2 Stanley Manne Children’s Research Institute, Simpson Querrey Biomedical Research Center, 303 E. Superior Street, Chicago, IL 60611 USA; 3 Cell and Molecular Physiology Department, Center for Translational Research and Education, Loyola University Chicago, Chicago, IL 60153 USA; 4 Department of Urology, Feinberg School of Medicine, Northwestern University, Chicago, IL 60611 USA; 5 Department of Biomedical Engineering, McCormick School of Engineering, Northwestern University, Evanston, IL 60208 USA; 6 Simpson Querrey Institute (SQI), Northwestern University, 303 East Superior Street, Chicago, IL 60611 USA

**Keywords:** Bladder augmentation, Bone marrow stem/progenitor cells, Non-human primate, Bladder smooth muscle, Bladder tissue regeneration, Stem cells, Medical research, Urology

## Abstract

Urinary bladder dysfunction can be caused by environmental, genetic, and developmental insults. Depending upon insult severity, the bladder may lose its ability to maintain volumetric capacity and intravesical pressure resulting in renal deterioration. Bladder augmentation enterocystoplasty (BAE) is utilized to increase bladder capacity to preserve renal function using autologous bowel tissue as a “patch.” To avoid the clinical complications associated with this procedure, we have engineered composite grafts comprised of autologous bone marrow mesenchymal stem cells (MSCs) co-seeded with CD34+ hematopoietic stem/progenitor cells (HSPCs) onto a pliable synthetic scaffold [poly(1,8-octamethylene-citrate-co-octanol)(POCO)] or a biological scaffold (SIS; small intestinal submucosa) to regenerate bladder tissue in our baboon bladder augmentation model. We set out to determine the global protein expression profile of bladder tissue that has undergone regeneration with the aforementioned stem cell seeded scaffolds along with baboons that underwent BAE. Data demonstrate that POCO and SIS grafted animals share high protein homogeneity between native and regenerated tissues while BAE animals displayed heterogeneous protein expression between the tissues following long-term engraftment. We posit that stem cell-seeded scaffolds can recapitulate tissue that is nearly indistinguishable from native tissue at the protein level and may be used in lieu of procedures such as BAE.

## Introduction

Urinary bladder dysfunction can arise from neurologic conditions including spina bifida (SB), spinal cord injury, traumatic brain injury, and military-based trauma^1,2^. For combat military personnel, bladder injury is often a result of penetrating trauma^3^. Between October 2001 through January 2008, US military personnel who served overseas were found to exhibit bladder injury at rate of 21.3% (or 189) of the recorded 887 who had unique genitourinary (GU) injuries^4^. In a study by Kronstedt et al. who analyzed the data set from the Department of Defense Trauma Registry spanning from January 2007 to March 2020, it was reported that 2584 combat soldiers injured during active duty required genitourinary surgery and 1090 (42%) required some type of bladder repair or reconstruction. Additionally, in a study of 530 veterans enrolled in Trauma Infectious Disease Outcomes Study (TIDOS), 89 patients acquired genitourinary injuries from deployment within the period of 2009–2014. Of the 89 patients, 19 (21%) had bladder injury and a majority (52.6%) of these patients had urinary tract infections^5^. The goal in managing a severely dysfunctional bladder includes preserving physiological function of the bladder so that urine can be stored under low pressure while maintaining its ability to void efficiently under volitional control. When conservative management fails, including the use of medicines or catheterization, surgical intervention in the form of urinary bladder diversion procedures or bladder augmentation enterocystoplasty (BAE) is often employed for the treatment of severe bladder conditions^6,7^. The ideal clinical outcome of these types of procedures is to increase bladder capacity and compliance by reducing intravesical pressure in order to protect renal function and improve on quality-of-life metrics^8^.

Bladder augmentation enterocystoplasty employs the use of autologous intestinal tissue including the ileum, gastric segments, or colonic segments. Unfortunately, this highly invasive surgical procedure poses unwanted long-term issues^1,6,8–10^. The ileum and colon promote the formation of bladder calculi at a rate of 3–52.5% and occurring as early as 5 months post-BAE^1,11,12^. The gastric segment is the last option and has a reduced risk of calculi but poses its unique complications such as hematuria-dysuria syndrome and increased risk of malignancy^9^. Additionally, other clinical issues arise over time including, excessive mucus production, electrolyte imbalances, and perforation can occur in all cases^13^. These issues are treatable in most cases but forces patients to perform intermittent self-catheterization to prevent bladder infection, calculi formation, and urinary tract infections. These issues can still arise even with ideal management, yet treatment efficacy varies patient to patient. Although BAE is marginally effective, the 10-year risk rate of secondary surgery is as high as 43.9% as demonstrated in spina bifida patients^14^.

As BAE is fraught with numerous short- and long-term clinical complications^17^ and there does not exist a quantitative assessment of the proteomic landscape of augmented tissue, here we describe the proteomic profiling of three unique grafts used for bladder augmentation. These include the current gold standard for BAE (ileum), small intestinal submucosa (SIS; a widely utilized biological scaffold)^15^, and the highly reproducible synthetic scaffold, poly(1,8-octamethylene-citrate-co-octanol) (POCO; also known as the CystoSTEM platform)^16^) within the context of a non-primate (baboon) bladder augmentation model. Both the SIS and POCO scaffolds were co-seeded with autologous bone marrow-derived mesenchymal stem cells (MSCs) and CD34+ hematopoietic stem/progenitor cells (HSPCs) prior to graft implantation^13,17,18^. Baboons underwent partial cystectomy and then independently grafted with either autologous ileum enterocystoplasty (**E**), cell-seeded SIS (**CS-SIS**), or cell-seeded POCO (**CS-POCO**). Native and regenerated (or ileum-augmented) bladder tissues were then collected and proteomic profiles of regenerated or ileum-augmented versus native bladder were analyzed and compared. This study is the first of its kind to demonstrate proteomic profiling in a large bladder tissue deficit baboon bladder augmentation model that bears significant anatomic and physiologic similarities to human counterparts. A regenerative engineering approach that can supplant the use of BAE would be highly beneficial so that the myriad of known clinical morbidities associated with BAE can be avoided as demonstrated by our CystoSTEM system. This would have significant impact on affected individuals so that better quality of life metrics can be attained.

## Materials and methods

### Baboon bladder augmentation surgical procedure

Baboon (Papio anubis) bladder augmentation procedures were performed by our group as previously described^13,16^. Briefly, a 50–65% partial bladder cystectomy was performed in animal cohorts and the bladder deficit was augmented with either autologous ileum (enterocystoplasty; **E**), cell-seeded (bone marrow derived, autologous MSCs and CD34+ HSPCs) biological scaffolds (**CS-SIS**), and cell-seeded biodegradable and elastomeric scaffolds (**CS-POCO**); n = 3 animals/group. Tissue-centric analyses utilized samples at 24 months (**CS-POCO**); 24 months (one animal at 27 months, **E**); 26–29 months (**CS-SIS**). In all groups, the isolation of regenerated or ileal tissue that was used for analysis was based upon permanent marking sutures placed at the native bladder/graft interface at the time of augmentation. This suture placement allowed us to distinguished native from scaffold (or ileal) augmented tissues. This was also accompanied by the visual inspection and further comparison between native and regenerated tissues as determined by the urological surgeon and experienced personnel. Physical differences between the two tissue types were apparent and notable. All animal procedures were performed in accordance with guidelines set forth and approved by the University of Illinois at Chicago Animal Care Committee (ACC) and the Northwestern University Institutional Animal Care and Use Committee (IACUC) and in accordance with ARRIVE guidelines.

### Protein and peptide purification

Stem cells were isolated and subsequently seeded onto their respective scaffolds as previously described^16^. Baboon bladder tissues were isolated immediately post-euthanization. This included both native bladder tissue (from the bladder base) and augmented bladder tissue (from the bladder dome). 50 mg of these tissues from each sample was homogenized in 1 ml lysis buffer containing 8 M urea, 1% SDS, in 50 mM HEPES pH 8.5, and HALT protease inhibitor cocktail (Thermo Fisher Scientific, Rockford, IL). The tissue extract was centrifuged at 3000 g for 15 min to eliminate tissue debris and the supernatant was transferred to a new tube. 200 ug of protein from each sample was purified from impurities and lipids by methanol-chloroform precipitation and resuspended in 6 M guanidine in 100 mM triethylammonium bicarbonate (TEAB). Proteins were reduced with 1 mM DTT and alkylated with 5 mM iodoacetamide, and were further diluted with 100 mM TEAB to minimize the guanidine hydrochloride concentration to less than 1.5 M before digestion with trypsin/lys-C protease mix, MS Grade, 1:50 ratio, (Thermo Fisher Scientific) overnight at 37 °C. The digest was then acidified with formic acid to a pH of ∼2–4 and desalted by using C18 HyperSep cartridges. The purified peptide solution was dried and quantified using the Micro BCA Protein Assay Kit (Thermo Fisher Scientific, Rockford, IL). An equal amount of peptide (∼50 μg) from each sample was then used for isobaric tandem mass tag (TMT-18plex) labeling as per the manufacturer’s instructions (Thermo Fisher Scientific).

### TMT-18plex labeling

TMT-18plex labeling on peptide samples was performed according to the manufacturer’s instructions (ThermoFisher Scientific). After two hours of incubation at room temperature, the reaction was quenched with hydroxylamine at a final concentration of 0.3% (v/v). Isobaric-labeled samples were then combined and lyophilized. The combined isobaric labeled peptide samples were then fractionated by Pierce High pH Reversed-Phase Peptide Fractionation Kit to eight fractions per the manufacturer’s protocol. Fractions were then dried using a speed vacuum concentrator and reconstituted in LC–MS sample buffer (5% acetonitrile, 0.125% formic acid) until LC–MS/MS analysis and concentration were assessed using Micro BCA. 1 µg of peptide was used for injection, and MS run was carried out using the following set up.

### MS/MS tandem mass spectrometry

Purified peptides, 1.0 ug each, were loaded onto a Vanquish Neo UHPLC system (Thermo Fisher Scientific) with a heated trap and elute workflow with a c18 PrepMap, 5 mm, 5 uM trap column (P/N 160,454) in a forward-flush configuration connected to a 25 cm Easyspray analytical column (P/N ES802A rev2) 2 uM, 100A, 75 um × 25 with 100% Buffer A (0.1% formic acid in water) and the column oven operating at 35 °C as described ^19^ [Peptides were eluted over a 240 min gradient, using 80% acetonitrile, 0.1% formic acid (buffer B), starting from 2.5% to 10% over 10 min, to 25% over 140 min, to 40% over 60 min, to 65% over 18 min, then to 99% in 5 min and kept at 99% for 7 min, after which all peptides were eluted]. Spectra were acquired with an Orbitrap Eclipse Tribrid mass spectrometer with FAIMS Pro interface (Thermo Fisher Scientific) running Tune 3.5 and Xcalibur 4.5 and using Real Time search filter (RTS) for MS3 triggering. For all acquisition methods, spray voltage set to 1900 V, and ion transfer tube temperature set at 300 °C, FAIMS switched between CVs of − 40 V, − 55 V, and − 70 V with cycle times of 1.0 s. MS1 detector set to orbitrap with 120 K resolution, wide quad isolation, mass range = normal, scan range = 400–1600 m/z, max injection time = 50 ms, AGC target = 300% microscans = 1, RF lens = 30%, without source fragmentation, and datatype = positive and centroid. Monoisotopic precursor selection was set to included charge states 2–6 and reject unassigned. Dynamic exclusion was allowed n = 1 exclusion for 40 s with 10 ppm tolerance for high and low. An intensity threshold was set to 5000. Precursor selection decision = most intense. MS2 settings include isolation window = 0.7, scan range = auto normal, collision energy = 30% CID, scan rate = turbo, max injection time = 35 ms, AGC target = 1 × 104, Q = 0.25. In MS3, an on-line real-time search algorithm (Orbiter) was used to trigger MS3 scans for quantification. MS3 scans were collected in the Orbitrap using a resolution of 50,000, scan range 100–500, notch number = 10, activation type HCD = 55%, maximum injection time of 200 ms, and AGC of 200%. Isobaric tag loss exclusion was set to TMT pro reagent^19^.

### MS/MS data analysis

Raw data were analyzed using Proteome Discoverer 2.5 (Thermo Fisher Scientific) using Sequest HT search engines. The data were searched against the Baboon UniProt Protein Sequence Database (Papio Anubis (species) Taxon ID9555). The search parameters included precursor mass tolerance of 10 ppm and 0.6 Da for fragments, allowing two missed trypsin cleavages, acetylation(+ 42.011 Da), Met-loss/ − 131.040 Da (M), and Met-loss + Acetyl (− 89.030 Da (M) as N-terminal dynamic modification and carbamidomethylation (Cys), TMTpro/ + 304.207 Da in any N-terminus, TMTpro/ + 304.207 Da (K) as a static modification. Percolator PSM validation was used with the following parameters: strict false discovery rate (FDR) of 0.01, relaxed FDR of 0.05, maximum ΔCn of 0.05, and validation based on q-value. Reporter ion quantitation was using the method 18-plex Tandem Mass Tag® of Proteome Sciences plc method implemented on the proteome discoverer software and general quantification settings used with following settings, Peptides to Use: Unique + Razor; Consider Protein Groups for Peptide Uniqueness set as True; Precursor Abundance Based On: Intensity; Normalization based on Total Peptide Amount; Scaling Mode set as none, Protein Abundance Caculation based on Top 3 Average Intensity, low abundance peptides were removed by filtering out proteins with less than 3 PSMs.

For class comparison, we used the intensity values obtained from the Proteome Discoverer software and exported them into BRB-array tools (Biometric Research Program) (vs. 4.2 D, National Cancer Institute) and normalized using quantile normalization. We used a stringent log ratio variation filter (50%) incorporated in the BRB array tools to remove proteins that do not change across all samples. Since the samples are compared between native and regenerated/grafted tissue from the same animal, we employed the Paired T-test (with random variance model) with following settings: random variance model parameters: a = 2.63561, b = 1.3768; Kolmogorov–Smirnov statistic = 0.00908; the exact Multivariate Permutations test was computed based on 1000 available permutations; and the maximum allowed local False Discovery Rate (FDR) of 0.05 was used to identify differentially expressed (DE) proteins.

For uncharacterized proteins or proteins with unknown function presented in the manuscript, the UniProt Accession number was searched against UniProt database https://www.uniprot.org/ and the NCBI Gene database https://www.ncbi.nlm.nih.gov/gene/. If required, more information was obtained with regards to protein identity by matching the amino acid sequence of the protein on the NCBI BLAST alignment program https://blast.ncbi.nlm.nih.gov/Blast.cgi.

### Data normalization

Data was normalized as per the Proteome Discoverer by calculating the total sum of the abundance values for each channel over all the peptides identified. The channel with the highest total abundance was considered as a reference, and correction was made for the abundance values in all other channels by a constant factor so that the total abundance in all channels was the same.

## Results

### Box plot distribution

**Figure 1:**
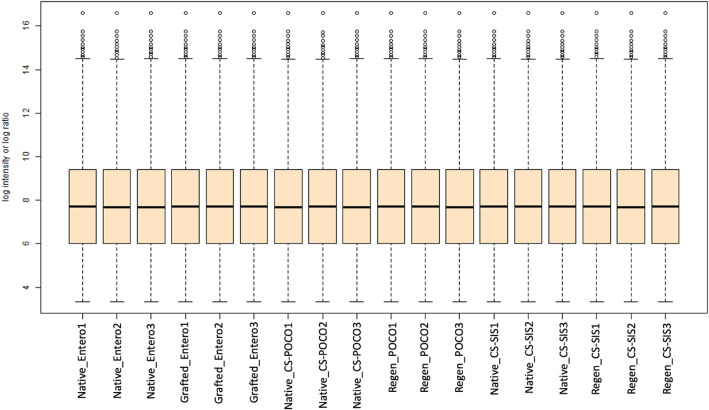
Normalized expression data of the 18 tissue samples in Box Plot Distribution format. Study groups consisted of **E**, **CS-POCO**, and** CS-SIS** (n = 3 animals each) and both the grafted or regenerated tissue was evaluated along with its donor-matched native tissue sample. The colored box indicated the Interquartile Range of 25–75% of the data range with a black line in the middle indicating the median. The outliers are single proteins that are shown for each sample.

In our study, we had examined the tissues collected from the native bladder tissue and compared against its own grafted tissue. Three baboons were used in each of the three study groups and two tissue samples from distinct bladder tissue anatomical locales (native or grafted areas) were obtained from each animal. These were either ileum-grafted (for **E** group) or regenerated (for **CS-POCO** and **CS-SIS**) and donor-matched native tissue samples. This resulted in a total of 18 tissue samples as shown in Fig. 1. The box plot was created to visualize the variation in abundances of mass spectrometric signals across different samples and conditions. As shown in the figure, the boxed area in each sample contained data that fell within Interquartile Range (IR), or 25–75% of the data range which includes data from Q1 to Q3, with the indicated median being at Q2. Any data points outside of the bars of the upper or lower region were single data points that are Q3 + 1.5IR or Q3 − 1.5IR, respectively. As shown in the figure, the IR for all 18 samples was similar and consistent across all samples indicating a uniform distribution of the data and suggested a tightly grouped result set. Since the median of the data was similar across all samples, there were minimal number of outliers and minimal variations variables or shifts amongst the samples.

### Volcano plots

Proteomic profiles of the regenerated (or grafted) bladder tissue versus the native bladder tissue for each animal were generated using our baboon bladder augmentation model. The data for the three animals were then averaged within each three groups (**E**, **CS-POCO,** and **CS-SIS**) of bladder augmentation. We analyzed a total of 5292 possible proteins and examined their expression patterns in the tissues within the aforementioned groups and determined the ratio of expression between the regenerated versus native for each tissue graft type.

**Figure 2:**
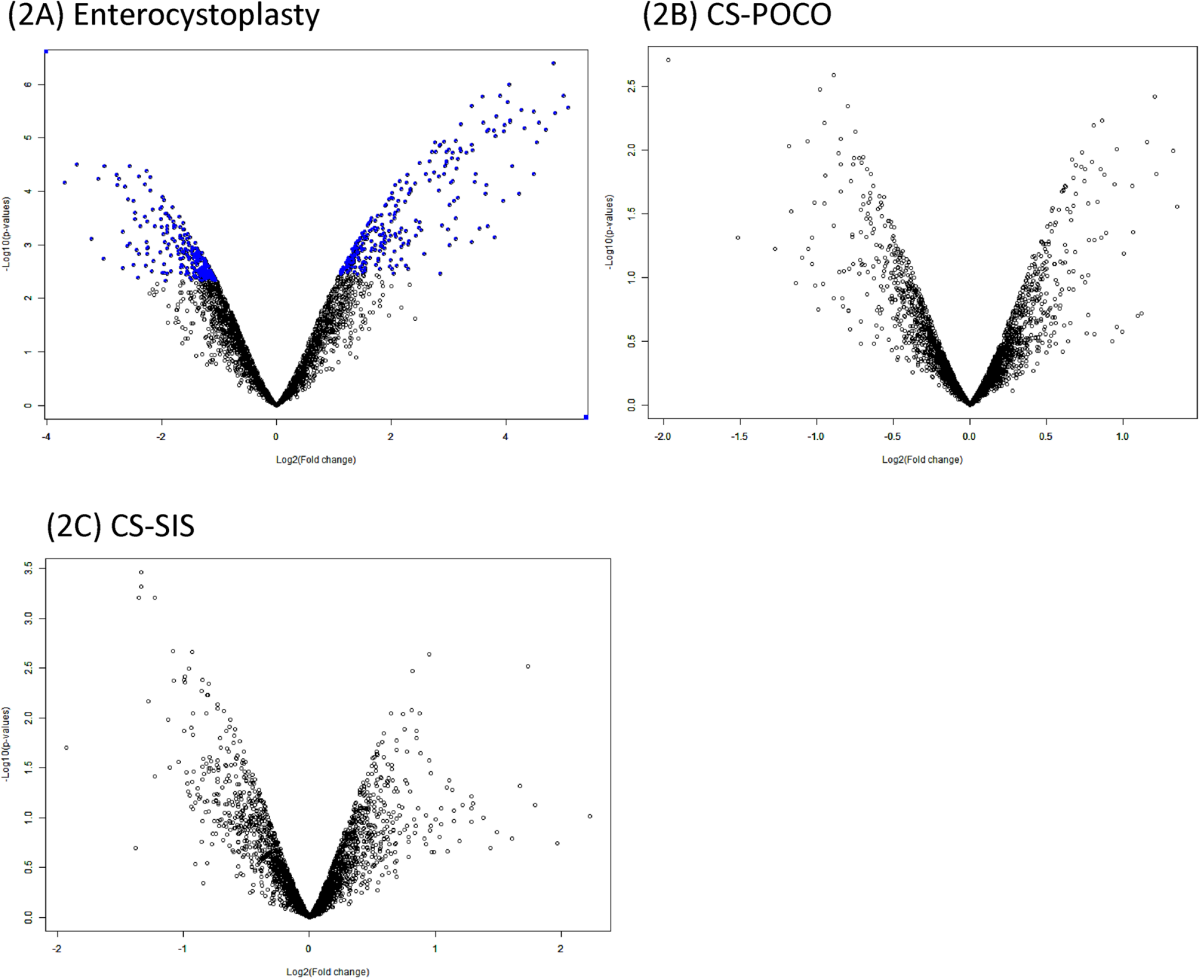
Volcano Plots demonstrating the differentially expressed protein distribution of the grafted or regenerated tissue versus the native tissue. Each protein is shown as a single point in the graph. Proteins that were differentially expressed in the grafted or regenerated tissue versus the native tissue at FDR > 0.05 are shown in blue color. (**A**)** E**: Grafted versus Native tissue showed numerous differentially expressed proteins in both grafted and native tissue with FDR > 0.05 shown in blue color. Both **CS-POCO**: Regenerated versus Native tissue (**B**) and** CS-SIS**: Regenerated versus Native (**C**) did not have any differentially expressed proteins that had FDR > 0.05. As shown in (**B**) and (**C**), those proteins had FDR < 0.05.

For visualization of differential protein expression pattern, the proteomics data from the three groups was represented in our volcano plots, with the *p*-value graphed against the log_2_ fold ratio. Figure 2A–C showed volcano plots of -Log_10_(*p*-value) versus Log_2_(Fold change) for the protein distribution in grafted (or regenerated) tissue to its native tissue in **E**, **CS-POCO**, and **CS-SIS**, respectively. For consideration for differential protein expression the allowed maximum FDR was 0.05. As indicated on the volcano plots, the areas where the proteins that were indicated as differentially expressed with the < 0.05 FDR criteria was marked in blue as shown in Fig. 2A in the **E** group. In Fig. 2B and C, **CS-POCO** and **CS-SIS** study groups, respectively, there were no differentially expressed protein in the either tissue.

### Differentially expressed proteins

As indicated in Fig. 2A volcano plot of the **E** group, of the 5292 proteins that were surveyed, 258 proteins were expressed at higher level in the native tissue compared to the **E** grafted tissue at FDR < 0.05. Conversely, 270 proteins had higher expression in the **E** grafted tissue than the native tissue at FDR < 0.05. Both of these Tables of differentially expressed proteins are provided as Supporting Information. The top 30 proteins that were overexpressed in the grafted tissue is shown in Table 1, while the top 30 overexpressed proteins in native tissue in Table 2. For each table, the identifying feature of the protein is the UniProt Accession number. Any uncharacterized proteins or proteins with unknown function were updated using information listed on the UniProt and GenBank Gene websites.

**Table 1:** Top 30 proteins overexpressed in Ileal-grafted (vs. Native) tissue.

UniquelD	Symbol	Protein name description	Entero-grafted/ native ratio	Parametric *p*-value	Local FDR
A0A096P102	ALDOB	Fructose-bisphosphate aldolase	33.86	0.0000027	0.000605
A0A096NIV1	TFF3	trefoil factor 3	32.1	1.60E−06	0.00046
A0A2I3MNY7	LGALS2	Galectin	29	0.0000034	0.000683
A0A096NGR8	DEFA6	defensin alpha 6	28.51	4.00E−07	0.000225
A0A096N2N1	CDH17	cadherin 17	25.86	6.90E−06	0.000998
A0A096N6M1	NTS	neurotensin	23.8	5.10E−06	0.000848
A0A8I5NAJ1		Peptidase S1 domain-containing protein	23.27	0.000012	0.00135
A0A096NXS7	REG3A	regenerating family member 3 alpha	22.44	4.76E−05	0.00296
A0A096N2A5	VIL1	villin 1	22.35	3.20E−06	0.000662
A0A096N861	SI	sucrase-isomaltase	20.02	6.50E−06	0.000967
A0A2I3LPH8	FABP1	fatty acid binding protein 1	19.34	3.00E−06	0.000639
A0A8I5NX92	FABP6	Cytosolic fatty-acid binding proteins domain-containing protein	18.8	0.0001101	0.00488
A0A096MX76	FABP2	fatty acid binding protein 2	17.21	3.41E−05	0.00244
A0A2I3NGN1	DDC	dopa decarboxylase	16.86	5.00E−06	0.000839
A0A8J8XMJ7	IFNAR2	Interferon alpha and beta receptor subunit 2	16.85	0.0000047	0.000812
A0A8I5R102		Regenerating family member 1 alpha	16.75	0.000005	0.000839
A0A096N2V6	CPS1	carbamoyl-phosphate synthase 1	16.68	1.00E−06	0.000361
A0A2I3N061	CLCA1	chloride channel accessory 1	16.28	2.10E-06	0.00053
A0A096MQK3	AOC1	amine oxidase, copper containing 1	15.69	5.80E−06	0.000909
A0A2I3MKQ3	AKR7A2	aldo–keto reductase family 7 member A2	15.55	7.40E−06	0.00104
A0A096NIV6	MEP1A	meprin A subunit alpha	15.38	0.0001518	0.00594
A0A8I5NV79	GPA33	glycoprotein A33	14.91	1.60E−06	0.00046
A0A096NB01	XDH	xanthine dehydrogenase	14.25	3.90E−06	0.000735
A0A2I3MUY0	EMILIN1	elastin microfibril interfacer 1	14.09	9.10E−06	0.00116
A0A096N291	ITLN1	intelectin 1	13.92	0.0007083	0.0159
A0A2I3NGG3	CHGA	chromogranin A	13.83	7.20E−06	0.00102
A0A096P5J1	KRT20	keratin 20	12.93	7.10E−06	0.00101
A0A096MQN7	RETNLB	resistin like beta	12.85	0.0004425	0.0117
A0A0A0MUV2	TJP3	tight junction protein 3	12.72	7.50E−06	0.00104
A0A096MW03	MYO1A	myosin IA	12.66	7.57E−05	0.00389

**Table 2:** Top 30 proteins overexpressed in Native (vs. Ileal-grafted) tissue.

UniquelD	Symbol	Protein name description	Native/Entero-grafted ratio	Parametric *p*-value	Local FDR
A0A8I5QZL2	PCP4	Purkinje cell protein 4	12.99	0.0000681	0.00377
A0A096NPC2	TRIM29	tripartite motif containing 29	11.11	3.10E−05	0.00246
A0A096NMC9	RASL12	RAS like family 12	9.09	0.000774	0.0154
A0A2I3NCR0	S100A2	Protein S100 (S100 calcium-binding protein)	8.33	0.0018127	0.0264
A0A096NZQ7	SBSPON	SMB domain-containing protein	8.33	0.000057	0.00342
A0A2I3LWV5	HPSE2	heparanase 2 (inactive)	7.69	3.39E-05	0.00258
A0A2I3M6J6	FHL1	LIM zinc-binding domain-containing protein	6.67	0.0000756	0.00399
A0A2I3N649	MPP2	membrane palmitoylated protein 2	6.67	5.73E−05	0.00343
A0A096NNR5	PTGIS	prostaglandin I2 synthase	6.67	4.83E−05	0.00313
A0A096N0J2	FAM180B	family with sequence similarity 180 member B	6.25	0.0027114	0.0343
A0A8I5NY61		Ig-like domain-containing protein	6.25	0.0010285	0.0184
A0A096MW08	CASQ2	calsequestrin 2	6.25	0.0005662	0.0128
A0A8I5NLI7	KLHL41	kelch like family member 41	6.25	8.15E−05	0.00416
A0A0A0MU45	NCCRP1	non-specific cytotoxic cell receptor protein 1 homolog (zebrafish)	5.88	0.0009448	0.0175
A0A0A0MW42	GNG7	G protein subunit gamma 7	5.88	0.0001422	0.00569
A0A096NUF7	FBLN5	fibulin 5	5.88	3.39E−05	0.00258
A0A096NLZ3	DUOX1	dual oxidase 1	5.56	0.0023881	0.0316
A0A096NLE0	SRL	sarcalumenin	5.56	0.0003295	0.00924
A0A8I5NBS2	EGFLAM	EGF like, fibronectin type III and laminin G domains	5.56	0.0002455	0.00778
A0A8I5N6C2	CDK5RAP1	CDK5 regulatory subunit associated protein 1	5.56	0.0001514	0.00589
A0A096NHA3	CRYM	crystallin mu	5.26	0.0040991	0.0453
A0A096N8N9	CHI3L1	chitinase 3 like 1	5.26	0.0014822	0.0232
A0A096N1Q1	MYL2	myosin light chain 2	5.26	0.0012537	0.0209
A0A096NHT4	PI16	peptidase inhibitor 16	5.26	0.0004518	0.0111
A0A096ND84	EFEMP2	EGF containing fibulin extracellular matrix protein 2	5.26	5.26E−05	0.00328
A0A096NXG3	TGM2	transglutaminase 2	5.00	0.0002979	0.00871
A0A2I3LFN3	CNDP1	carnosine dipeptidase 1	5.00	7.39E−05	0.00395
A0A096N6P6	MMP7	matrix metallopeptidase 7	4.76	0.0024404	0.032
A0A0A0MWS8	TNNI3	Troponin I, cardiac muscle (Cardiac troponin I)	4.76	0.0014769	0.0231
A0A8I5NES5	DGKG	diacylglycerol kinase gamma	4.76	0.001166	0.0199

In Table 1, the top 10 proteins with the highest abundance ratio in the **E** grafted tissue compared to native tissue included Fructose-bisphosphate aldolase (ALDOB) (abundance fold ratio of 33.86, *p*-value of 0.0000027), trefoil factor (TFF3), (abundance fold ratio of 32.1, *p*-value of 1.60E−06), Galectin (LGALS2) (abundance fold ratio of 29, *p*-value of 0.0000034), defensin alpha 6 (DEFA6) (abundance fold ratio of 28.51, *p*-value of 4.00E−07), cadherin 17 (CDH17) (abundance fold ratio of 25.86, *p*-value of 0.6.90E−06), neurotensin (NTS) (abundance fold ratio of 23.8, *p*-value of 5.10E−06), Peptidase S1 domain-containing protein (abundance fold ratio of 23.37, *p*-value of 0.000012), regenerating family member 3 alpha (REG3A) (abundance fold ratio of 22.4, *p-*value of 4.76E−05), Villin-1 (VIL1) (abundance fold ratio of 22.35, *p*-value of 3.20E−06), and sucrase-isomaltase (SI) (abundance fold ratio of 20.02, *p*-value of 6.50E−06).

**Figure 3:**
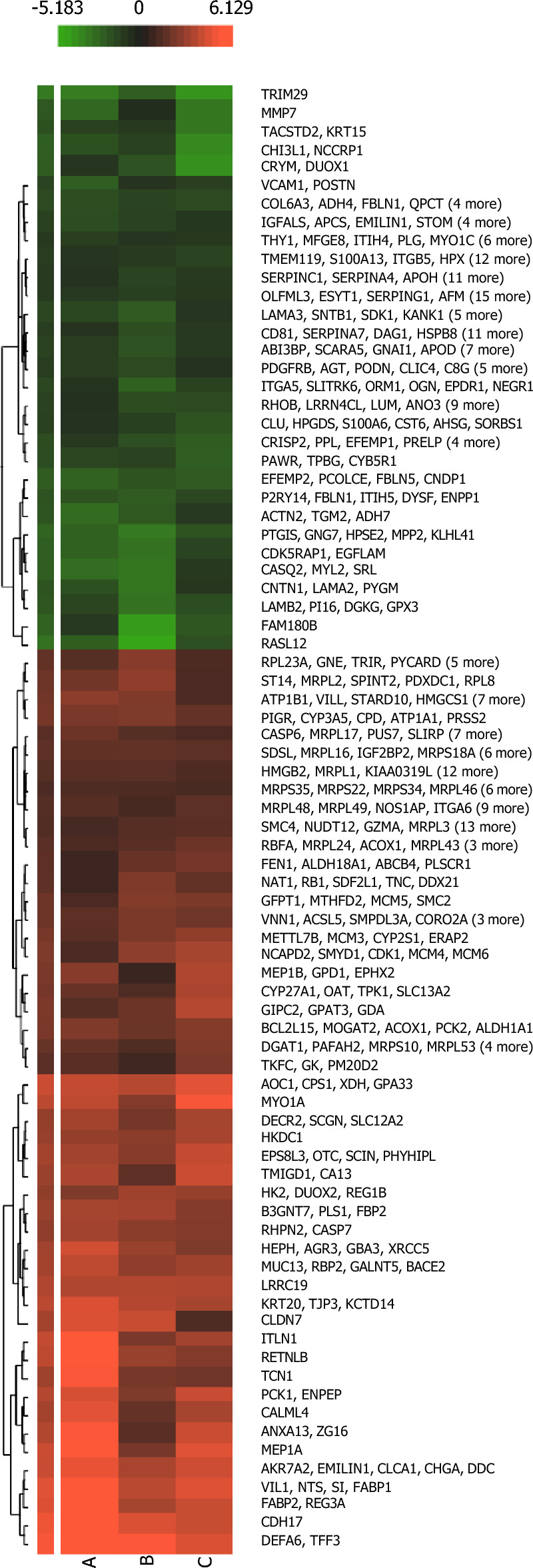
Heat Map Distribution of the proteins differentially expressed in paired grafted-ileal tissue vs its matched native tissue. Columns A, B, and C represent animals 1–3 from the **E** group.

In Table 2, the top 10 differentially expressed proteins having the highest abundance ratio in the native tissue compared to ileum-grafted tissue included Purkinje cell protein 4 (PCP4) (abundance fold ratio of 12.99, *p*-value of 0.0000681), Tripartite motif containing 29 (TRIM29) (abundance fold ratio of 11.35, *p*-value of 3.10E−05), RAS like family 12 (RASL12) (abundance fold ratio of 9.09, *p*-value of 0.000774), Protein S100 (S100 calcium-binding protein) (S100A2) (abundance fold ratio of 8.33, *p*-value of 0.0018127, SMB domain-containing protein (SBSPON) (abundance fold ratio of 8.33, *p*-value of 0.000057), heparanase 2 (inactive)-HPSE2 (abundance fold ratio of 7.69, *p*-value of 3.39E−05), LIM zinc-binding domain-containing protein (FHL1) (abundance fold ratio of 6.67, *p*-value of 0.0000756), membrane palmitoylated protein 2 (MPP2) (abundance fold ratio of 6.67, *p*-value of 5.73E−05, prostaglandin I2 synthase (PTGIS) (abundance fold ratio of 6.67, *p*-value of. 4.83E−05), and family with sequence similarity 280-member B (FAM180B) (abundance fold ratio of 6.25, *p*-value of 0.0027114). The Heat Map demonstrating differentially expressed proteins is illustrated in Fig. 3. SI Figs. 1, 2, 3 and 4 provide data with respect to class/protein distribution regarding Biological Processes: DE protein lists were analyzed using the ShinyGo web application using default parameters, with *p*-value cutoff (FDR) < 0.05, and top 30 pathways to show Biological processes, molecular function, KEGG pathways and Reactome pathways enriched in the DE in list (Supporting Information)^20^. An exhaustive list of proteins expressed within enterocystoplasty grafts (ileum and native tissue) is provided as SI (accompanying Excel spreadsheet). Complete bladder histological and physiological assessments pertaining to the study can be found in our previous study^16^.

## Discussion

Delineation of a protein signature during urinary bladder tissue regeneration would provide invaluable data where specific proteins could be studied as stand-alone molecules or pools that could be used to propel bladder tissue regeneration. However, this has presented itself with numerous obstacles that include data capturing tools to animal models that may (or may not) faithfully represent their human counterparts. One alternative would be the real-time, protein expression data capture during active tissue regeneration and this may become a reality as technology advanced. Until then, we report the proteomic profile of bladder augmented tissue compared to its native bladder tissue in a long-term study using a unique baboon bladder augmentation model. Our established baboon model was utilized to recapitulate aspects of human anatomy and physiology which share many significant similarities. Three bladder augmentation conditions were evaluated that included the ileal-graft, consisting of autologous grafted ileum, the CystoSTEM platform, and the stem cell seeded biological scaffold SIS. The cells used for co-seeding of the scaffold included autologous donor-matched bone marrow MSCs and CD34+ HSPCs. Over the duration of our in vivo studies, these bone marrow-derived cells demonstrated robust tissue regenerative prowess of a major bladder defect as a direct result of our cystectomy. To demonstrate and analyze protein expression in these settings, we utilized a cut off of FDR < 0.05 for the differentially expressed proteins in native tissue vs regenerated (or grafted) tissues. Using these criteria, we have found that the “gold standard” bladder augmentation enterocystoplasty procedure that is clinically used yielded the most differentially expressed proteins at a total of 528 proteins. 270 of these proteins were expressed at higher levels in the grafted tissue versus native while 258 proteins expressed at higher levels in native versus grafted tissue. The proteins with the highest fold level of differential protein expression were in the ileal grafted tissue where a total of 10 proteins had abundance fold ratio of ranging from 20 to 33.86. This result is not unexpected but further demonstrates the protein heterogeneity and incongruences between bladder and ileal tissue. Yet ileum and other gastrointestinal segments are still routinely used today and serve as pseudo-bladder tissue in an attempt to recreate functional bladder tissue. One highly, overexpressed protein in the ileal graft was DEFA6, Defensin alpha 6. It had the highest differential fold ratio of 28.51. DEFA6 is highly expressed in the secretory Paneth cells that reside in the small intestine^21,22^. DEF6A protects the intestinal mucosa and defends against invasion of viruses and bacteria by forming fibrils and nanonets that encompasses pathogens^23^. It has however also reported that DEFA6 is also highly expressed in colorectal cancer (CRC) cell lines and patient samples^24^. By knocking down DEF6A expression via shRNA in cancer cells, Jeong et al. observed significantly inhibited cell growth, migration, and invasion in cancer cells in vitro, and inhibited tumorigenesis in vivo compared to control cells^24^. Furthermore, it was determined that high DEF6A expression to be a strong prognostic indicator for CRC as high expression of DEF6A was observed in 51.4% (or 181/352) primary colorectal cancer tissue samples with high correlation to poor prognosis^23^. In a study by Husmann et al., it was revealed that patients who undergone BAE had an incidence of 5–6% to develop bladder cancer when approaching their fifth decade^25^. It is worth noting that whether or not that DEF6A was observed to be highly expressed in cancer tissue, that investigation into this protein maybe warranted to determine its potential role in tumorigenesis in bladder tissue post BAE. For the proteins with higher expression in the native tissue versus the grafted tissue included 2 proteins with abundance fold ratio greater than 10, which included PCP4 (Purkinje cell protein 4) and TRIM29 (Tripartite motif containing 29).

In our study using the cell seeded grafts, the CystoSTEM platform or **CS-SIS**, the proteomic analysis did not reveal any differentially expressed proteins using the FDR < 0.05 criteria. Whole bladder tissue proteomic profiling data demonstrate that the cell-seed grafts are similar to native bladder tissue at the protein level following lengthy in vivo regeneration.

Our current data demonstrate that overall bladder tissue regeneration is driven through stem-cell based signaling derived most likely from the MSC/CD34+ HSPC seeded-combination. We have previously shown a synergistic effect with this MSC/CD34+ HSPC combination in vivo that may be dependent on specific molecules found within the Wnt family of proteins^17^. These include Wnt10a, TCF3/4, FZD5, and CTNNB1 and we speculate that the interplay between this network of molecules directs bladder tissue regeneration on multiple fronts. Ongoing studies will attempt to delineate a mechanism of action to provide a better understanding of the regenerative landscape. Finally, the use of autologous bone marrow-derived stem and progenitor cells did not illicit an immune response, nor did the scaffold which is biocompatible and non-toxic as we have previously described^16,17^.

Although protein profiling data for the **CS-SIS** and **CS-POCO** grafts were very similar, their long-term physiological outputs coupled with tissue regenerative metrics can be quite disparate as we have described^16^. As SIS is a decellularized biological material derived from porcine intestinal tissue, its extreme pliability contributes to its lack of structural rigidity subsequently resulting in major mechanical properties mismatches when compared to native baboon (or human as they are similar in modulus) bladder tissue. This can lead to poor bladder tissue regeneration accompanied by poor bladder capacity recovery eventually leading to renal distress and dysfunction over time. Thus the implementation of the CystoSTEM system would provide a seamless transition in support of bladder tissue regeneration.

### Supplementary Information

Supplementary Information 1.

Supplementary Figures.

## Data Availability

The datasets generated and/or analyzed during the study are available in the Center for Computational Mass Spectrometry, Computer Science and Engineering, UCSD (MassIVE MSV000093971).

## Abbreviations

BAE: Bladder augmentation enterocystoplasty
CD34+ HSPC: CD34+ hematopoietic stem/progenitor cells
CS: Cell-seeded
POCO: Poly(1,8-octamethylene-citrate-co-octanol)
SIS: Small intestinal submucosa
