# Supplementary material for: Proteomic profiling of regenerated urinary bladder tissue in a non-human primate augmentation model
Source: Sci Rep. 2024 Jul 9;14:15757. doi: 10.1038/s41598-024-66088-9 (PMC11231185; doi:10.1038/s41598-024-66088-9)
Supplement: Supplementary file 2 — Supplementary Figures. [file 41598_2024_66088_MOESM2_ESM.docx]

**Supporting Information**

**SI Figure 1.** Biological Processes- Distribution of genes that are involved in an assortment of functions that are pivotal in tissue reorganization following insult. Of particular interest are processes related to wound healing regulation and its responses. Within the **E** group of animals, there is a wide distribution of genes involved in organizational aspects of tissue remodeling as one would anticipate when employing ileum as a pseudo-bladder patch.

**SI Figure 2.** KEGG Pathway Analysis- Within the context of our baboon bladder augmentation model, it was unexpected to observe multiple tissue hits with regards to genes related cardiomyopathy. Future studies will try to determine the potential role of this set of genes in our system.

**SI Figure 3.** Molecular Function- An examination of functional/enzymatic genes revealed high retinoid binding expression. As noted in SI Figure 2, retinol is enriched >6-fold. As both genes are intimately involved a number of biological processes, we speculate that these two genes are involved with cell growth, differentiation, and maintenance that is required of the epithelium of the ileal graft that maintains constant cellular turnover.

**SI Figure 4.** Protein Reactome Analysis- High mitochondrial activity is noted genes encompassing various mitochondria-based processes. This is to be expected as the graft itself is highly, metabolically active as in its native anatomical position. This may contribute to poor clinical manifestations including mucus production that can serve as a source of continued infections.
